# Supplementary material for: Limitations of hormonal and clinical markers in predicting GnRH agonist trigger success in polycystic ovary syndrome: A critical reappraisal
Source: PLoS One. 2025 Oct 23;20(10):e0332813. doi: 10.1371/journal.pone.0332813 (PMC12548884; doi:10.1371/journal.pone.0332813)
Supplement: Attachment — Submitted filename: letter-plos.docx [file pone.0332813.s002.docx]

Dear Editor,

We sincerely thank the Academic Editor and the reviewers for their thoughtful and constructive comments. We have carefully considered each point and revised the manuscript accordingly. Below, we provide a detailed response to every comment, indicating how and where changes were made in the revised manuscript. We hope that the revisions and clarifications adequately address all concerns and improve the quality and clarity of our work.

# Response to Reviewers

## Reviewer #1:

**Comment 1:** "Abstract: The short Background is missing."

**Author Response:**

We thank the reviewer for this insightful comment. In accordance with the suggestion, we have revised the abstract to include a concise background section. The revised version now briefly explains the clinical relevance of GnRH agonist triggers in PCOS patients and the rationale for investigating predictors of oocyte yield. This addition helps contextualize the study aim for readers from the outset.

**Revised Abstract Opening:**

“**Background:** Polycystic ovary syndrome (PCOS) is associated with altered hypothalamic-pituitary-ovarian function, which may affect the success of GnRH agonist triggers used during IVF. Identifying reliable predictors of oocyte yield in these patients remains a clinical challenge.”

We hope this revision meets the reviewer’s expectations and improves the clarity and completeness of the abstract.

## Comment 2: "Manuscript content: Rationale is missing which should be part of the Introduction."

**Author Response:**

We appreciate the reviewer’s comment regarding the importance of clearly presenting the rationale in the Introduction section. To address this, we have added a concise explanation highlighting the current uncertainty in identifying reliable predictors of oocyte yield in PCOS patients undergoing GnRH agonist trigger. This paragraph now establishes the unmet clinical need and positions our study as a focused attempt to address this gap.

**Added Text in the Introduction (last paragraph):**

While the use of GnRH agonist triggers offers clear clinical benefits for PCOS patients undergoing IVF, accurately predicting suboptimal oocyte yield remains a significant challenge. Identifying reliable pre-trigger predictors could enhance patient selection and improve treatment outcomes. Nevertheless, the existing literature presents inconsistent findings, and studies focusing exclusively on PCOS cohorts are notably scarce.

 Determining predictors for poor responses in PCOS is certainly a challenge and I applaud the authors for their attempt, even if it was not the outcome they may have hoped for. I would suggest another limitation is the use of MPA prior to stim start instead of utilizing laboratory values to ensure patients were at the same phase of cycle (ie early follicular) vs. a "random start."
We appreciate the reviewer’s insightful comment regarding the need to further elaborate on the clinical implications of post-trigger hormonal assessment and the potential limitations in intervention once the trigger has been administered.

In response, we have added the following paragraph to the Discussion section to clarify our clinical rationale and emphasize the importance of pre-trigger evaluation:

“Considering that corrective options are limited once a suboptimal hormonal response is detected after the trigger, it becomes increasingly important to focus on the factors within our control prior to triggering. In our clinical practice, therefore, comprehensive evaluation of pre-trigger parameters—including AFC, baseline LH, BMI, and patient history—is emphasized to anticipate the likelihood of a suboptimal response. This proactive strategy is particularly relevant for PCOS patients, in whom altered gonadotropin dynamics may not always translate into predictable post-trigger outcomes.”

We believe this addition strengthens the clinical relevance of our findings and addresses the reviewer’s concern.

**Response to Reviewer 2:**

We sincerely thank the reviewer for the valuable observation regarding the use of MPA prior to stimulation and the potential implications for cycle phase standardization.

We agree that initiating stimulation based on laboratory-confirmed early follicular phase could offer tighter control over the baseline hormonal milieu. However, in patients with PCOS—who often experience oligo/anovulation—this approach may not always be practical. In our study, we chose to administer MPA to induce withdrawal bleeding, thereby ensuring a standardized and timely initiation of ovarian stimulation across participants.

In addition to facilitating synchronization, the use of MPA may have also contributed to the suppression of elevated basal LH levels—a common feature in PCOS—which could otherwise interfere with the interpretation of pre-trigger hormonal profiles. This rationale has now been clarified and acknowledged as both a methodological choice and a potential limitation in the revised Discussion section.

We appreciate the reviewer’s thoughtful input, which helped improve the clarity of our design considerations.
